# Eosinophilic peritonitis and nephrotic syndrome in Kimura’s disease: a case report and literature review: Eosinophilic peritonitis in Kimura’s disease

**DOI:** 10.1186/s12882-020-01791-z

**Published:** 2020-04-17

**Authors:** Bingxin Yu, Zhikai Yang, Di Song, Zi Wang, Damin Xu, Suxia Wang, Lin Nong, Fude Zhou, Jie Dong

**Affiliations:** 1 Renal Division, Department of Medicine, Peking University First Hospital, Beijing, China; 2 Institute of Nephrology, Peking University, Beijing, China; 3 Key Laboratory of Renal Disease, Ministry of Health of China, Beijing, People’s Republic of China; 4 Laboratory of Electron Microscopy, Pathological Centre, Peking University First Hospital, Beijing, China; 5 Department of Pathology, Peking University First Hospital, Beijing, China

**Keywords:** Kimura’s disease, Eosinophilic peritonitis, Nephrotic syndrome, Peritoneal dialysis

## Abstract

**Background:**

Eosinophilic peritonitis is a relatively rare entity. Kimura’s disease is a rare chronic inflammatory disorder of unknown etiology, characterized by subcutaneous nodules mainly in the head and neck region, regional lymphadenopathy and occasional involvement of kidney. There is currently no report of eosinophilic peritonitis in Kimura’s disease.

**Case presentation:**

A 44-year-old Chinese man presented with abdominal distention, nausea, vomiting and edema in lower limbs for 1 month. Laboratory data showed elevated eosinophils in peripheral blood and ascites, nephrotic syndrome with progressively renal dysfunction, and elevated IgE. Ultrasonography of lymph nodes showed multiple lymphadenopathy in bilateral inguinal regions. Surgical excision was performed for one of the enlarged lymph nodes and histopathology revealed diagnosis of Kimura’s disease. Renal biopsy indicated focal segmental glomerulosclerosis (FSGS) and acute tubulointerstitial nephritis with infiltration of eosinophils in renal interstitium. The patient was prescribed with oral prednisolone therapy (30 mg/day), and underwent continuous ambulatory peritoneal dialysis (CAPD). The peripheral and peritoneal eosinophil count decreased rapidly and normalized within 2 days. Forty-five days after prednisolone therapy, partial remission of nephrotic syndrome and decrease of serum creatinine were achieved while peritoneal dialysis dosage had decreased. Inguinal lymph nodes gradually shrunk in size. The overall conditions remain stable afterwards.

**Conclusions:**

This rare case highlighted the clinical conundrum of a patient presenting with eosinophilic peritonitis, lymphadenopathy, nephrotic syndrome and renal failure associated with Kimura’s disease. The remarkable eosinophilia, pathology of lymph node and kidney, as well as significant response to steroids should guide towards the diagnosis.

## Background

Eosinophilic peritonitis is a relatively rare entity. The etiology of eosinophilic peritonitis is multifactorial, mostly being reported in patients with eosinophilic gastroenteritis, parasitic and fungal infections, hypereosinophilic syndrome and less common diseases including malignancy, hematological disorders, inflammatory bowel disease, autoimmune disease and peritoneal dialysis [1–3]. Kimura’s disease is a rare chronic inflammatory disorder of unknown etiology, characterized by subcutaneous nodules mainly in the head and neck region, regional lymphadenopathy and occasional involvement of the major salivary glands and kidney. It is often accompanied by peripheral eosinophilia and markedly elevated serum IgE levels [4]. There is currently no report of eosinophilic peritonitis in Kimura’s disease. Here is a case of eosinophilic peritonitis manifested by Kimura’s disease accompanied with nephrotic syndrome.

## Case presentation

A 44-year-old man was admitted to the hospital and reported abdominal distention, with positive shifting dullness and edema in lower limbs. He also complained of acid regurgitation, nausea, and vomiting for 1 month, without fever, abdominal pain, rash, or oliguria. He had been prescribed with penicillin for a week without remission. The patient had history of edema and was diagnosed as nephrotic syndrome 22 years ago but didn’t undergo renal biopsy. He was treated with 60 mg/day prednisone for almost 1 year and tapered to cease until negative proteinuria, while intermittently taking moderate dose of prednisone afterwards whenever urine dipsticks showed positive proteinuria by September 2018. However, he didn’t monitor blood routine examination and serum creatinine. He denied history of asthma, atopy and family history of kidney diseases. The patient was a non-smoker and denied alcohol ingestion. On physical examination, his blood pressure was normal, and cardiopulmonary examination was sound. There was one palpable lymph nodal mass in each side of inguinal region, both were non-tender and firm in consistency, with normal overlying skin.

On admission, laboratory data showed nephrotic syndrome with peripheral eosinophilia at 2.1 × 10^9^/L (24.3%) and elevated IgE (171 IU/L). Stool analysis for parasitic eggs and parasites didn’t show abnormalities. Abdominal ultrasonography demonstrated a maximum 10 cm depth of ascites. Computed tomography (CT) revealed multiple mesentery exudation, thickened peritoneum, and ascites, without hepatosplenomegaly, intra- and extra-hepatic bile ducts dilation and intra-abdominal lymphadenopathy. Diagnostic abdominal paracentesis revealed translucent yellow fluid. The white cell count in the ascitic fluid was 200/mm^3^, with 14% eosinophils. Chemical analysis indicated transudate with serum-acites albumin gradient (SAAG) of 15.4 g/L. Ascitic fluid smears and cultures for bacterial, tuberculous and fungus were negative. Serum immunoglobulin (IgG) level was low, while IgA, IgM, C3 and C4 level was normal. Serum and urine immunofixation electrophoresis showed monoclonal IgG λ, with normal free κ/λ ratio in serum. Bone marrow cytology, histopathology and flow cytometry showed no abnormality.

**Fig. 1:**
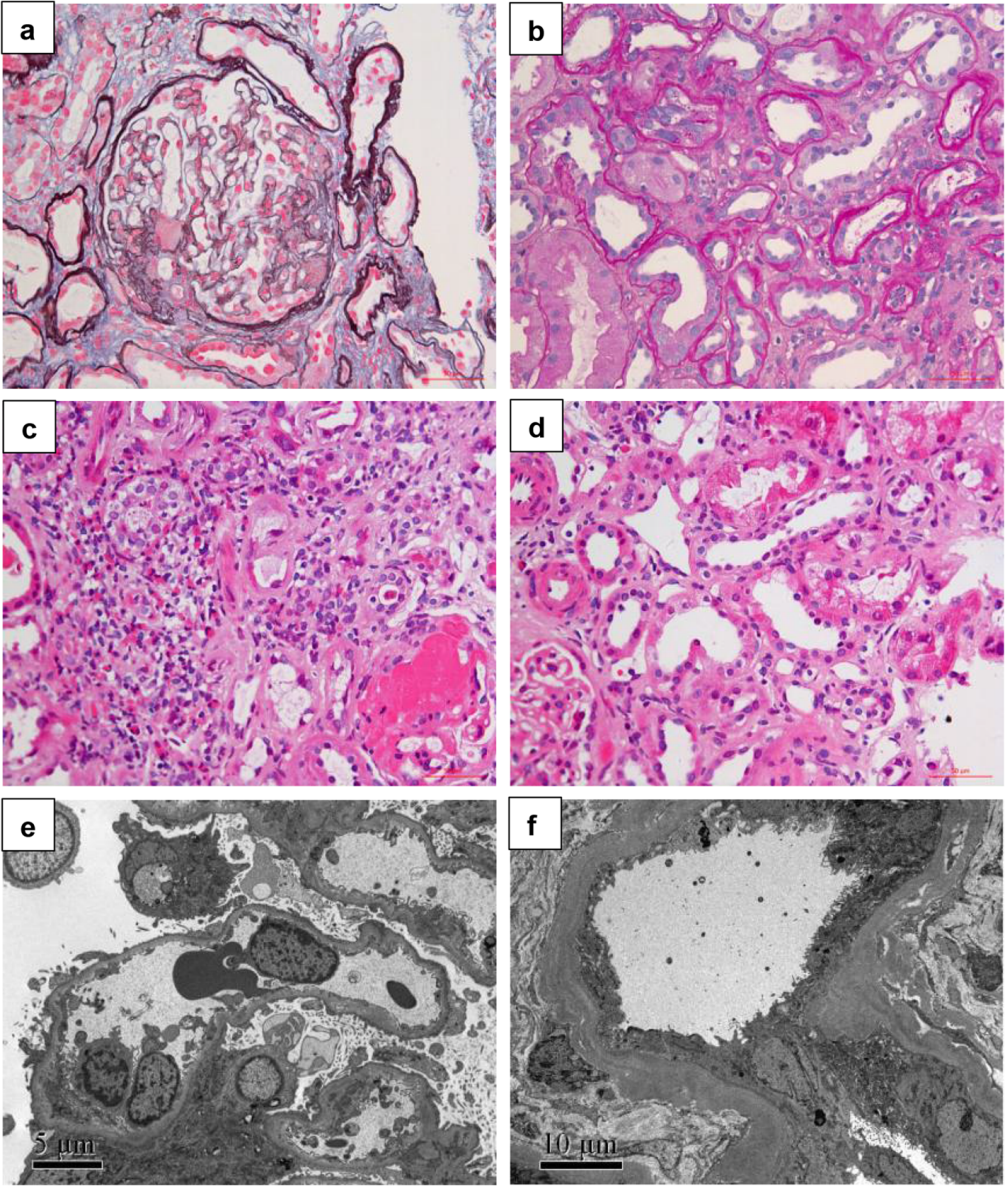
Renal pathology features. Renal specimen showed focal segmental glomerulosclerosis, acute tubularinterstitial nephritis with red blood cell and protein casts in tubules and eosinophilic infiltration in renal interstitium. Electron microscope showed no electron dense deposit. (**a**: PAS + MASSON × 400; **b**: PAS × 200; **c**: HE × 400; **d**: HE × 400; **e**: EM × 5000; f: EM × 3000)

Serum albumin was 18 g/L, 24-h proteinuria was 13.9 g/day (urine volume 1450 ml). Serum creatinine (Scr) progressively elevated from 472 μmol/L to 612 μmol/L within 8 days before admission to hospital, and the peak Scr was 844 μmol/l on 14th day after admission. Serum IgG4 was in the normal range. Anti-PLA2R, anti-dsDNA, ANCA and anti-GBM antibody were all negative. Abdominal ultrasonography demonstrated relatively small size of kidney. Due to the progressively increasing Scr, peritoneal dialysis was performed to relieve uremia symptoms and drainage ascites. Ultrasound-guided renal biopsy was performed. Twenty-eight glomeruli were included in the specimens for light microscopy. Of them, 17 glomeruli were ischemic global sclerosis, 9 glomeruli had segmental glomerulosclerosis with adhesion. Patchy interstitial inflammation of lympoytes, mononuclear cells along with eosinophilils. Immmunofluorescence showed focal IgM ++, C3 +++ in mesangium and segmental sclerosis of glomeruli. The electron microscopy demonstrated diffuse effacement of podocytic foot processes without electron dense deposits. Final pathological diagnosis was focal segmental glomerulosclerosis (FSGS) and acute tubulointerstitial nephritis. There were no signs for IgG4 related disease or monoclonal gammopathy of renal significance (MGRS) (Fig. 1).

**Fig. 2:**
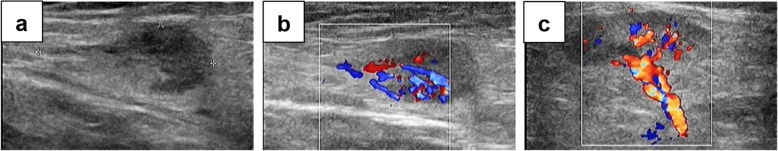
Ultrasonography of bilateral inguinal regions of the patient. Ultrasonography showed multiple lymph nodes enlargement, with the largest measuring about 2.6 × 0.9 cm (**a**). The lymph node was rich in blood vessels (**b**). Blood vessels entering lymph node were clearly depicted (**c**)

**Fig. 3:**
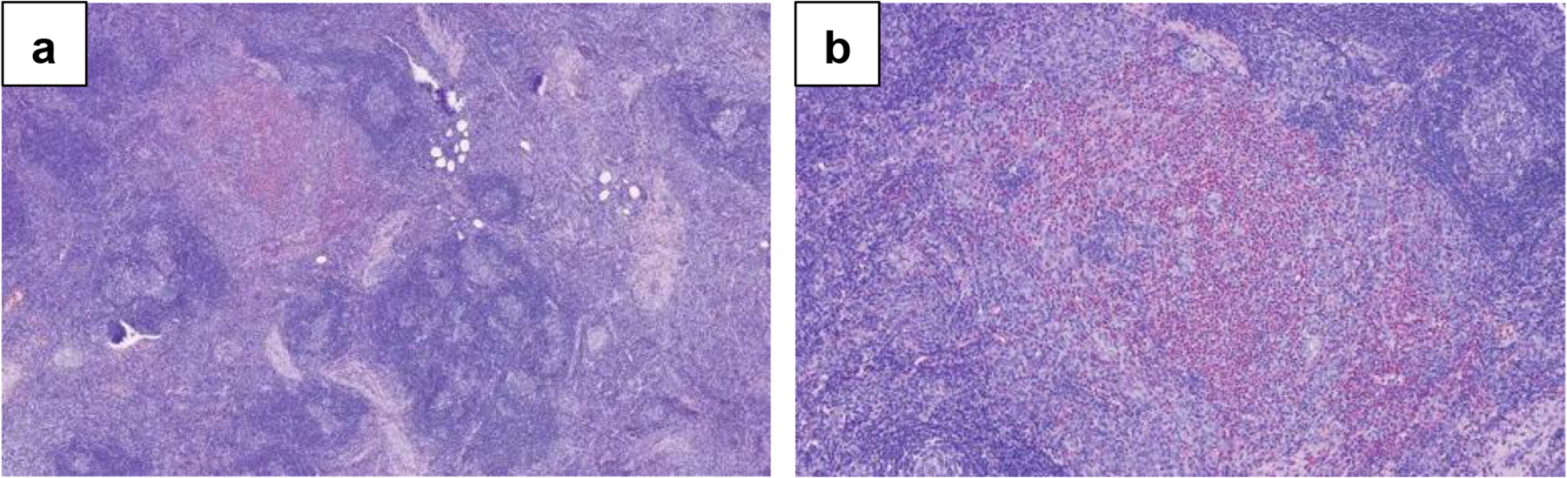
Lymphnode pathology features. Prominent germinal centers and interfollicular areas with massive infiltration of eosinophils and eosinophilic microabscesses (**a**: H&E × 40). Eosinophilic microabscesses and interfollicular vascular proliferation (**b**: H&E × 100)

Ultrasonography of lymph nodes showed multiple lymphadenopathy in bilateral inguinal regions, with the largest measuring about 2.6 × 0.9 cm in the left inguinal region, and 2.7 × 0.8 cm in the right inguinal region (Fig. 2). Screening for HIV, Syphilis, cytomegalovirus (CMV), epstein-barr virus (EBV) and **e**valuation of interferon-gamma release assay for tuberculosis didn’t show abnormalities. Screen for toxoplasmosis was not performed, since he didn’t have epidemiological history of toxoplasmosis. Surgical excision was performed for one of the enlarged lymph nodes (1.5 cm in diameter) in the right inguinal region. Microscopic examination of H&E-stained sections showed preserved lymph node architecture, follicular and interfollicular hyperplasia, as well as increased eosinophils with formation of eosinophilic microabscesses within the germinal centers (Fig. 3). The histopathology of lymph node didn’t show caseous granuloma or other infectious features, such as toxoplasmic lymphadenitis. Hence, a diagnosis of Kimura’s disease was established with relevant clinical details and specific histopathological features.

Based on the clinical data, the eosinophilic peritonitis, nephrotic syndrome and lymphadenopathy was considered to be associated with systematic involvement of Kimura’s disease. The patient was prescribed with oral prednisolone therapy (30 mg/day), and underwent continuous ambulatory peritoneal dialysis (CAPD). Anti- infective therapy wasn’t administered. The peripheral and peritoneal eosinophil count decreased rapidly and normalized within 2 days. Forty-five days after prednisolone therapy, 24-h proteinuria reduced to 2.3 g/d (Urine volume 1500 ml), and serum albumin level increased to 31 g/L. Serum creatinine decreased gradually to around 350 μmol/L while peritoneal dialysis dosage had decreased from 4500 ml/d to 3000 ml/d. It’s noteworthy that residual urea clearance (Kt/V) gradually increased to 2.77. Inguinal lymph nodes gradually shrunk in size, with the largest measuring about 1.9 × 0.6 cm in the left inguinal region, and 2.4 × 0.5 cm in the right inguinal region. Prednisolone was tapered to 25 mg/d and peritoneal dialysis dosage decreased from 3000 ml/d to 1500 ml/d after 60 days. The overall conditions maintained stable afterwards (Table 1).

**Table 1:** Time-course changes in 24-h proteinuria, serum albumin, serum creatinine, Kt/V, Ccr and lymph node size of bilateral inguinal regions according to prednisolone therapy

	admission	0	15 days	45 days	75 days	135 days
Prednisolone dosage, mg/d	/	30	30	30	25	25
24-h proteinuria, g/d	13.9	5.8	6.1	2.3	3.4	2.4
Serum albumin, g/l	18	18	19	31	35.9	39.3
Serum creatinine, umol/l	829	559	528	344	367	354
Peritoneal dialysis dosage, ml/d	0	4500	3000	3000	1500	1500
Kt/V renal	/	/	1.29	2.77	2.10	4.25
Kt/V peritoneal	/	/	0.51	0.75	0.29	0.34
Ccr renal, l/week per 1.73 m^2^	/	/	24.69	51.83	39.54	81.92
Ccr peritoneal, l/week per 1.73 m^2^	/	/	0.51	0.83	0.35	9.49
Left maximal long axes (mm)	/	21	26	19	16	20
Left maximal short axes (mm)	/	8	9	6	5	5
Right maximal long axes (mm)	/	24	27	24	20	24
Right maximal short axes (mm)	/	7	8	5	5	7

Kt/V renal was estimated via the concurrent 24-h urine urea excretion. Kt/V Peritoneal was estimated via 24-h dialysate urea excretion and the serum urea concentration. Ccr. creatinine clearance rate; Ccr peritoneal was estimated from the 24-h dialysate creatinine excretion and the serum creatinine concentration at the completion of the collection. Ccr renal was estimated as the average of renal creatinine and urea clearance (Ccr + Curea)/2

Note: prednisone was tapered to 25 mg/d at 60 d. Peritoneal dialysis dosage decreased from 4500 ml/d to 3000 ml/d at 10 d; peritoneal dialysis dosage decreased from 3000 ml/d to 1500 ml/d at 60 d;

## Discussion and conclusions

We present a case of elevated eosinophils in peripheral blood and ascites, nephrotic syndrome with progressively renal dysfunction and lymphadenopathy. Several diseases presenting with eosinophilia, nephrotic lesions and lymphadenopathy must be differentiated from Kimura’s disease, including acute interstitial nephritis, lymphoma, polyneuropathy, organomegaly, endocrinopathy, monoclonal protein and skin syndrome (POEMS), IgG4 related disease. In this case, lymph node pathology and renal biopsy plays an important role in distinguishing it from other diseases. Another important differential diagnosis of Kimura’s disease is angiolymphoid hyperplasia with eosinophils (ALHE), which is also called as epithelioid hemangioma (EH) nowadays. EH/AHLE and Kimura’s disease can share similar clinical and morphological features, and they were once considered different stages of the same disease. Peripheral eosinophilia, elevated IgE, lymphadenopathy and nephrotic syndrome is more common seen in Kimura’s disease than EH/ALHE. Difference of histopathological features helps to distinguish this entity. Histologically, florid lymphoid follicles with germinal center formation, eosinophilic infiltrates, eosinophilic microabscesses, and eosinophilic folliculolysis are salient features of Kimura’s disease, the histiocytoid/ epithelioid-endothelial cells are characteristic of ALHE/EH [5, 6].

Eosinophilic peritonitis is a relatively rare entity defined as the presence of > 100 eosinophils/mm^3^, or > 10% eosinophils of the total non-erythrocyte count [7]. A recent systematic review reported in patients with eosinophilic peritonitis, 74% had eosinophilic gastroenteritis, 10% parasitic and fungal infections, 7% hypereosinophilic syndrome and 9% patients with less common diseases (eosinophilic pancreatitis, chronic eosinophilic leukemia, myelofibrosis, T-cell lymphoma, Churg Strauss syndrome, systemic lupus erythematosus, familial paroxysmal polyserositis and Ménétrier’s disease) [1–3]. However, there is currently no report of Kimura’s disease with eosinophilic peritonitis among general population. Therapy with corticosteroids results in resolution in almost all patients with eosinophilic ascites [1]. In this case, eosinophil counts in ascites correlated with peripheral blood eosinophil counts, both decreased to normal after receiving corticosteroids therapy, similar to previous studies [7].

Nephrotic syndrome has been reported as a complication of Kimura’s disease, including membranous nephropathy, minimal change disease, diffuse proliferative glomerulonephritis, mesangial proliferative glomerulonephritis and membrane proliferative glomerulonephritis [8], with a few FSGS cases [9–11]. Steroid monotherapy has been used for Kimura’s disease and FSGS with varying degrees of success [9–12]. In this case, the patient was steroid-sensitive on remission of eosinophils, eosinophilic peritonitis, lymph nodes and partial remission of renal damage, which was clinically considered to be associated with systematic involvement of Kimura’s disease, however without stronger evidence, and needs long-term follow-up. Association between Kimura’s disease and renal involvement remains unclear. Previous studies suggest that the predominance of Th2 and Tc1 cells and certain cytokines including IL-5 as an eosinophil-active cytokine and IL-10 may contributes to the pathogenesis of Kimura’s disease, but further research is warranted [13–15].

This patient had elevated IgG λ in serum and urine in the absence of plasma cell or lymphoid malignancy or end organ damage, therefore monoclonal gammopathy of undetermined significance (MGUS) was determined [16]. Though there is currently no report of Kimura’s disease with MGUS, lymphatic proliferative disease is cautious to be ruled out.

Nowadays, there is no consensus regarding the optimal treatment for Kimura’s disease. Systemic corticosteroids was chosen for this patient because of the associated nephrotic syndrome.

In conclusion, the rare case highlighted the clinical conundrum of a patient presenting with eosinophilic peritonitis, lymphadenopathy, nephrotic syndrome and renal failure associated with Kimura’s disease. Prompt treatment led to early remission, however kidney function was not completely recovered***.*** Advantage of the study is timely diagnosis and proper differential diagnosis, the remarkable eosinophilia, pathology of lymph node and kidney, as well as significant response to steroids should guide towards the diagnosis.

## Data Availability

All data related to this case report are within the manuscript.

## Abbreviations

CAPD: Continuous ambulatory peritoneal dialysis
CT: Computed tomography
SAAG: Serum-acites albumin gradient
Ig: Immunoglobulin
Scr: Serum creatinine
MGRS: Monoclonal gammopathy of renal significance
MGUS: Monoclonal gammopathy of undetermined significance
ALHE: Angiolymphoid hyperplasia with eosinophils
EH: Epithelioid hemangioma
FSGS: Focal segmental glomerulosclerosis;
